# Comparative evaluation of surface characteristics of dentinal walls with and without using plastic finishing file

**DOI:** 10.4103/0972-0707.66719

**Published:** 2010

**Authors:** Smita Singh, Neeraj Nigam

**Affiliations:** Department of Conservative and Endodontics, Darshan Dental College and Hospital, Ranakpur Road, Loyara, Udaipur, Rajasthan, India

**Keywords:** Dentinal debris, hand protaper, hand profile, hand hero shaper, plastic F file and stereomicroscope

## Abstract

**Aim:**

The aim of this *in vitro* research is to evaluate the debris present on the dentinal walls after instrumentation in mesiobuccal root of maxillary molar with Hand Protapers, Hand Profiles, Hand Hero Shapers, and finishing the dentinal walls with and without rotary plastic files using stereomicroscope.

**Materials and Methods:**

Sixty freshly extracted human maxillary first molar teeth were divided into two groups of 30 teeth each (group A and B). Both the groups were divided into three subgroups of 10 teeth each (group A had subgroups A1, A2, and A3) and (group B had subgroups B1, B2 and B3). In group A, all the 30 canals were subjected to cleaning and shaping with three different instruments system and kept unfinished. This group was named as unfinished group. In group B, all the 30 canals were subjected to cleaning and shaping with three different instruments system followed by finishing with plastic files. This group was named as finished group. The crown of each tooth was sectioned at the cementoenamel junction. Canals were prepared following conventional principles of crown down technique. After splitting the roots longitudinally, the dentinal debris of each root canal was evaluated in three areas (coronal, middle and apical thirds of the root) by means of numerical evaluation scale, using a stereomicroscope.

**Results:**

Stereomicroscopic evaluations showed that there was no significant difference in the debris scores between the subgroups when the canals were instrumented with hand Protaper, hand Profile and hand Hero Shaper in all the thirds. There was no significant difference in scores between the unfinished group and the finished group in the coronal third but significant differences were seen in middle third and apical third.

**Conclusions:**

After instrumentation with different hand instruments, the use of plastic finishing files showed cleaner canal walls.

## INTRODUCTION

Successful root canal treatment depends on many factors and among them the prime importance is of the cleaning ability of any root canal instrument in the removal of micro organisms, infected dentine, organic and inorganic tissue by shaping and dissolution.[1]

The aim of root canal instrumentation is to create a tapered shape to allow effective volume of irrigation and a three-dimensional obturation.[2] Canals prepared with stainless steel instruments were only superficially cleaned and much of the pulp tissue was not removed. Stainless steel files have also been shown to create canal aberrations, such as ledges, perforations, zips and elbows.[3,4] To eliminate some of the short comings of these traditional endodontic instruments, nickel-titanium (Ni-Ti) instruments have been developed. Most of the new systems incorporate instruments with a taper greater than ISO standard .02 design.[4,5] Besides variation in taper, nickel-titanium instruments are characterized by different cross-sections and blade design.[4]

Ni-Ti root canal instruments have become an important part of an endodontic armamentarium. Most of these newly introduced systems have been investigated with regard to their shaping ability in curved canals in extracted teeth. Studies have shown that Ni-Ti instruments can effectively produce a well-tapered root canal form sufficient for obturation, with minimal risk of transporting the original canal.[5–10] Moreover, these investigations have shown that the different Ni-Ti instruments produce inconsistent results and this variation in the debris removal efficiency of these instruments may result from variation in flute designs. Obviously, instruments with sharp cutting edges seem to be superior to those having radial lands.[11]

In the last decade, three new Ni-Ti instruments with sharp cutting edges were introduced, but little information exists about their cleaning ability. Consequently, the aim of this investigation is to compare the cleaning efficacy (residual debris) after preparation of root canals with Hand ProTaper instruments (Dentsply Maillefer, Switzerland), Hand Profile (Dentsply Maillefer, Switzerland) and Hand HeroShapers (Micro Mega, USA).

Recently, the advancement of polymer science and technology have allowed for the development of a plastic rotary endodontic finishing file (Plastic Endo, LLC, USA). This plastic rotary endodontic file provides a time and cost saving advantage over sonic or ultrasonic instrumentation.

## MATERIALS AND METHODS

### Criteria for selection of teeth and storage

Sixty freshly extracted human maxillary first molar teeth were collected from the outpatient department of oral and maxillofacial surgery. The collected teeth were washed under tap water to remove blood stains and soft tissue tags. The teeth were stored in normal saline until further use. Teeth were radiographed in a buccolingual direction with RVG and care was taken to exclude teeth: open apices, severely curved and dilacerated roots, presence of root fillings, internal resorption, and calcified canals.

### Method of sectioning of teeth at CEJ

The crown of each tooth was sectioned at the cementoenamel junction. The coronal portion was removed with a water-cooled double-faced diamond disk operated at low speed. Then all the roots were inspected for canal patency with # 10 K-file.

### Method of canal preparation till no. 15 K file

The working length for all groups was obtained by measuring the length with #08/#10 K-file at the apical foramen minus 1 mm. The canals were enlarged till #15 K file. After each instrument, the root canal was flushed with 3% NaOCl and EDTA (Glyde).

### Method of group division

- For stratified randomization of the sample, the specimens were assigned to two groups of 30 teeth each (group A and B). Both the groups were divided into three subgroups of 10 teeth each (group A had subgroups A1, A2, and A3) and (group B had subgroups B1, B2 and B3). In group A, all the 30 canals were subjected to cleaning with 3three different instrument systems and kept unfinished. This group was named as unfinished group. In group B, all the 30 canals were subjected to cleaning with three different instrument system followed by finishing with plastic files. This group was named as finished group.
- Cleaning of the canals was done with hand Protaper, hand Profile and hand Hero Shapers for subgroup A1, A2 and A3, respectively, which remained unfinished at the end. Cleaning of the canals was done with hand Protaper, hand Profile and hand Hero Shapers for subgroup B1, B2 and B3, respectively, which were finished with plastic files at the end.
- All the 60 canals of groups A and B were instrumented till #20 apical sizes [Table 1].

**Table 1:** Data for debris score for unfinished and finished group

Group	Thirds	Coronal third score					Middle third score					Apical third score					Total score				
	Instrument	1	2	3	4	5	1	2	3	4	5	1	2	3	4	5	1	2	3	4	5
Unfinished	A1 Protaper	1	2	2	5	0	0	0	6	4	0	0	0	3	6	1	1	2	11	15	1
	A2 Profile	0	1	8	1	0	0	4	5	1	0	1	2	5	2	0	1	7	18	4	0
	A3 H.shaper	0	4	6	0	0	0	0	8	2	0	0	5	4	1	0	0	9	18	3	0
Finished	B1 Protaper	2	4	3	1	0	6	3	0	1	0	4	3	1	1	1	12	10	4	3	1
	B2 Profile	2	5	2	1	0	3	5	1	1	0	4	4	0	1	1	9	14	3	3	1
	B3 H.shaper	3	5	1	1	0	4	4	2	0	0	8	0	2	0	0	15	9	5	1	0

### Instrumentation with Protaper instruments

Protaper instruments were used in subgroup A1 and B1. Instruments were used following the conventional principles of crown down technique for this system till apical size #20.

### Instrumentation with profile instruments

Profile instruments were used in subgroup A2 and B2. Instruments were used following the conventional principles of crown down technique for this system till apical size #20.

### Instrumentation with Hero Shaper instruments

Hero Shaper instruments were used in subgroup A3 and B3. Instruments were used following the conventional principles of crown down technique for this system till apical size #20.

### Instrumentation with plastic F files

Once all the instrumentations were completed, the plastic rotary endodontic finishing file was used for finishing in finished group i.e. group B. Prior to placement of the F File (Plastic Endo, LLC, USA), the canal was filled with NaOCl. The F File was inserted in an electric slow speed hand piece that was set at 300 rpm. The working length was set on the F File with a rubber stopper, and then the file was placed passively into the canal and circumferentially worked along the dentinal walls with cyclic axial motion (up and down). The F File was used in the canal for approximately 15 sec, and then the canal was flushed with NaOCl and dried. Only one F File was instrumented per tooth and was discarded after single use.

### Method of evaluation

All the canals were completed by one operator. After preparation, all the canals were flushed with normal saline and dried with absorbent paper point. The evaluations were recorded for debris. The cleanliness of each root canal was evaluated in three areas (coronal, middle and apical thirds of the root) by means of numerical evaluation scale.[12] Debris includes dentinal chips, pulp remnants; particles loosely attached to the canal wall and retained irrigant (EDTA).

### Scoring criteria

**Figure 1:**
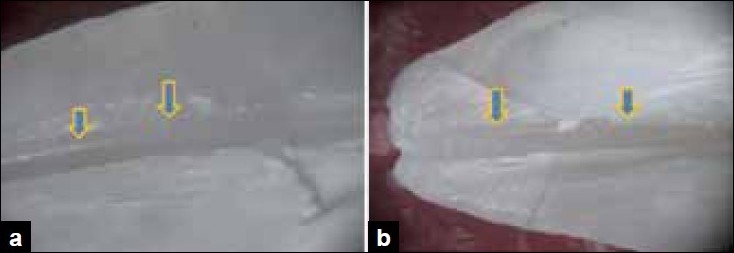
a) Stereomicroscopic study shows the middle third section for score 1- clean canal wall. b) Stereomicroscopic study shows the middle third section for score 1- clean canal wall.
- Score 2: few small conglomerulations on the canal wall [Figure 2a and b].

- Score 1: clean canal wall, scanty debris particles present, if any [Figure 1a and b].

**Figure 2:**
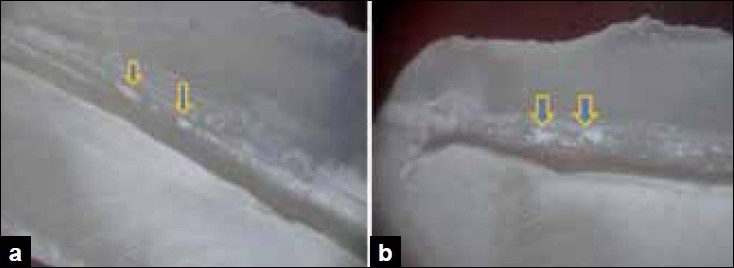
a) Stereomicroscopic study shows the middle third section for score 2- few small conglomerulations. b) Stereomicroscopic study shows the apical third section for score 2- few small conglomerulations
- Score 3: many conglomerulations, less than 50% of the canal wall covered [Figure 3a and b].

**Figure 3:**
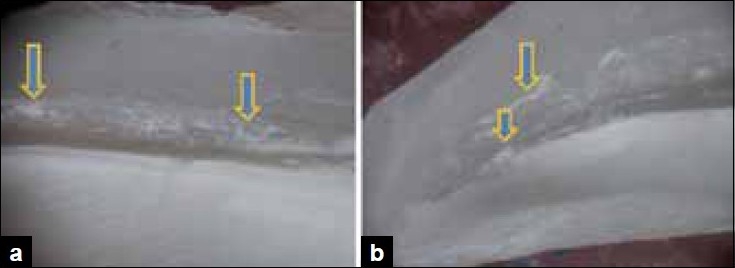
a) Stereomicroscopic study shows the middle third section for score 3- many conglomerulations, less than 50% of the canal walls covered. b) Stereomicroscopic study shows the apical third section for score 3- many conglomerulations, less than 50% of the canal walls covered
- Score 4: more than 50% of canal wall covered [Figure 4a and b].

**Figure 4:**
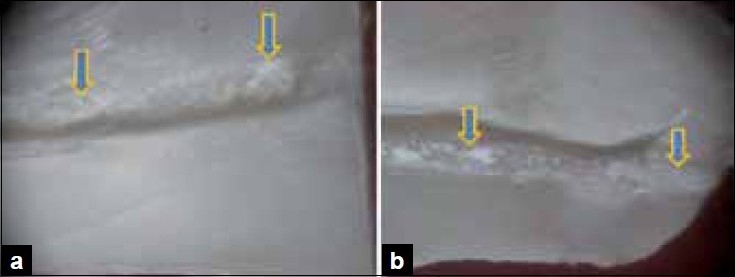
a) Stereomicroscopic study shows the middle third section for score 4- more than 50% of the canal walls covered b) Stereomicroscopic study shows the apical third section for score 4- more than 50% of the canal walls covered
- Score 5: completely or nearly complete covering of the canal wall by debris [Figure 5a and b].

**Figure 5:**
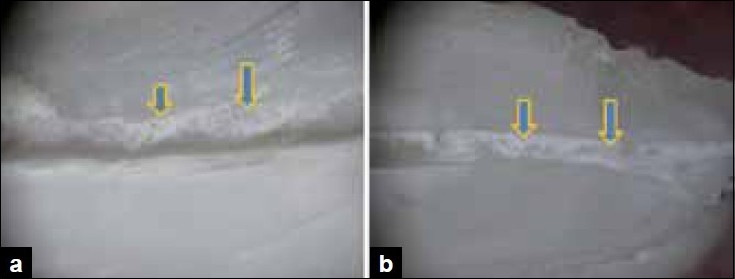
a) Stereomicroscopic study shows the middle third section for score 5- completely or nearly complete covering of the canal wall by debris b) Stereomicroscopic study shows the apical third section for score 5- completely or nearly complete covering of the canal wall by debris

### Method of longitudinal sectioning

Splitting (L.S) of the canals was done longitudinally with water-cooled double-faced diamond disk operated at low speed. Sectioned samples were evaluated under Stereomicroscope (Olympus SZX, Japan). Each thirds of the root were selected and visualized under Stereomicroscope, illuminated with Nikon 100 W optical fibre. The images were captured at 20 X original magnification.

## RESULTS

The data established for scoring the debris were recorded and analyzed statistically. One way ANOVA was used for comparison of the two groups. The level of statistical significance was set at *P*<0.05.

Inference: Table 2 shows that there was no significant difference in the scores between the subgroups in all the thirds when the instruments were compared. There was no significant difference in scores between the unfinished group and the finished group in coronal thirds (*P*=0.137) and in middle thirds (*P*=0.004), but significant difference in scores were seen in apical thirds (*P*=0.007).

**Table 2:** Mean debris score and SD of all the instrument systems for unfinished and finished group

Portion	Group	Score	1	2	3	4	5	*P* value
Coronal	Unfinished	Mean	0.33	2.33	5.33	2.00	0.00	0.137(NS)
		SD	0.58	1.53	3.06	2.65	0.00	
	Finished	Mean	2.33	4.67	2.00	1.00	0.00	
		SD	0.58	0.58	1.00	0.00	0.00	
Middle	Unfinished	Mean	0.00	1.33	6.33	2.33	0.00	0.004(S)
		SD	0.00	2.31	1.53	1.53	0.00	
	Finished	Mean	4.33	4.00	1.00	0.67	0.00	
		SD	1.53	1.00	1.00	0.58	0.00	
Apical	Unfinished	Mean	0.33	2.33	4.00	3.00	0.33	0.007(S)
		SD	0.58	2.52	1.00	2.65	0.58	
	Finished	Mean	5.33	2.33	1.00	0.67	0.67	
		SD	2.31	2.08	1.00	0.58	0.58	
Total	Unfinished	Mean	0.67	6.00	15.67	7.33	0.33	0.000(S)
		SD	0.58	3.61	4.04	6.66	0.58	
	Finished	Mean	12.00	11.00	4.00	2.33	0.67	
		SD	3.00	2.65	1.00	1.15	0.58	

## DISCUSSION

One of the most important objectives during root canal instrumentation is the removal of vital and/or necrotic pulp tissue, infected dentine debris in order to eliminate most of the micro organism from the root canal system.[2,13]

In this study, the cleaning efficiency of the different instruments was assessed using debris presence. Debris is defined as dentinal chips, and residual vital or necrotic pulp tissue attached to the root canal wall, which in most cases is infected.[12] Thus debris might prevent the efficient removal of micro organism from the root canal system. Moreover, debris may occupy part of the root canal space and thus may also prevent complete obturation of the root canal.[14]

Although the use of antibacterial irrigants is recommended in combination with chelating agents in order to remove debris as well as the inorganic/ organic smear layer,[12,15–18] NaOCl and EDTA were used as irrigants in the present study. NaOCl would appear as the best available canal irrigant because of its antibacterial and organic tissue-dissolving properties,[19,20] but it is not possible to remove the smear layer with NaOCl.[17,18,21,22] Nevertheless, considering the major objective of the present investigation (to compare the cleaning effectiveness of three instrumentation technique under identical conditions), a combined irrigation was used. As it has been shown recently by several authors[12,17,18] that EDTA (Glyde) containing chelating agents may be partially responsible for effective cleaning of canal walls after instrumentation with files, it has been taken into consideration that the cleaning efficiency of the two instruments evaluated in the present study might be further improved using a combination of NaOCl and EDTA.[12,17,18]

In the present study, the cleaning efficiency of three instrumentation sequences was examined on the basis of a separate numerical evaluation scheme for debris, by means of a stereomicroscope evaluation for the coronal, middle, and apical portions of the canals.[12,23] With three instrumentation techniques, partially uninstrumented areas with remaining debris were found in all canals sections. This finding has also been described by others.[8,12,24]

During the late 1980s and early 1990s, significantly modified endodontic instruments were designed, tested and marketed. Studies have shown that nickel-titanium instruments can effectively produce a well-tapered root canal form sufficient for obturation, with minimum risk of transporting the original canal.

There was no significant difference in the scores between the subgroups when the canals were instrumented with the Protaper, the Profile and the Hero Shaper in all the thirds. There was no significant difference in scores between the unfinished group and the finished group (*P*=0.137) in coronal thirds. There was a significant difference in scores between the unfinished group and the finished group (*P*=0.004) in middle thirds. There was a significant difference in scores between the unfinished group and the finished group (*P*=0.007) in apical thirds. There was a significant difference in scores between the unfinished group and the finished group (*P*=0.000) in the total canal area.

The improved result could be because of the unique file design with a diamond abrasive embedded into a non-toxic polymer that enables the new endodontic polymer-based rotary finishing file to agitate sodium hypochlorite and remove remaining dentinal wall debris without further enlarging the canal. From this research, it is evident that plastic files promise a brilliant future in finishing of root canal walls.
